# Potential biomarkers for active renal involvement in systemic lupus erythematosus patients

**DOI:** 10.3389/fmed.2022.995103

**Published:** 2022-12-01

**Authors:** Lu Xiao, Wei Xiao, Shudian Lin

**Affiliations:** ^1^Department of Rheumatology, Hainan General Hospital, Hainan Affiliated Hospital of Hainan Medical University, Haikou, China; ^2^Department of Respiratory, Hainan General Hospital, Hainan Affiliated Hospital of Hainan Medical University, Haikou, China

**Keywords:** systemic lupus erythematosus, lupus nephritis, biomarker, SLEDAI, transcription factor

## Abstract

**Objective:**

This study aimed to identify the key genes related to active renal involvement in patients with systemic lupus erythematosus (SLE).

**Methods:**

Microarray datasets were downloaded from the Gene Expression Omnibus (GEO) database. Differentially expressed genes (DEGs) between SLE patients with active renal involvement and those who did not have active renal involvement were identified by R software. Hub genes were identified using protein–protein interaction networks. The relationships between the expression levels of identified hub genes and SLEDAI were subjected to linear correlation analysis. The diagnostic accuracy of the hub genes was evaluated with the area under the curve of the receiver operating characteristic curve (ROC-AUC). Transcription factors (TFs) were predicted. The expression levels of different hub genes and histopathological patterns were also examined.

**Results:**

A total of 182 DEGs were identified. Enrichment analysis indicated that DEGs were primarily enriched in neutrophil degranulation, neutrophil activation involved in immune response and neutrophil activation. The expression levels of 12 identified hub genes were verified. Ten of the 12 hub genes were positively associated with SLEDAI. The combination model of DEFA4, CTSG, RETN, CEACAM8, TOP2A, LTF, MPO, ELANE, BIRC5, and LCN2 had a certain diagnostic accuracy in detecting renal involvement with high disease activity in SLE patients. The expressions of five predicted TFs were validated by GSE65391 dataset.

**Conclusion:**

This work explored the pathogenesis of renal involvement in SLE. These results may guide future experimental research and clinical transformation.

## Introduction

Systemic lupus erythematosus (SLE) is an autoimmune disease with clinically heterogeneity; it predominantly affects young women (1). Renal involvement can be seen in up to 70% of patients with SLE and is the most critical predictor of the morbidity and mortality of SLE. Manifestations of renal involvement can vary from macroscopic proteinuria and hematuria to nephrotic syndrome, cast excretion, and end-stage renal disease (2). Considering that the severe complications may be caused by renal involvement, and the treatment options for renal involvement are limited, novel biomarkers that can monitor and predict the progression of renal involvement need to be identified (3).

Bioinformatics is a branch of computer science that is widely used to explore promising biomarkers to improve disease diagnosis and treatment at the genome level (4–6). Numerous bioinformatic studies have demonstrated different abnormal expression levels of genes associated with the development of lupus nephrits (LN). In 2021, Zhimin Chen et al. downloaded kidney biopsy sequencing data to identify LN hub genes and differentially expressed genes (DEGs). They discovered six valuable biomarkers (HLA-DMA, HLA-DPA1, HLA-DPB1, HLA-DRA, IL10RA, and IRF8) that are strongly correlated with LN diagnosis and prognosis (7). In addition, a group of researchers used single-cell RNA sequencing to investigate the immune cell landscape in the kidneys of patients with LN. They found evidence that the local activation of B cells was correlated with an age-associated B-cell signature; a clear interferon response was observed in most cells. Two chemokine receptors, namely, CXCR4 and CX3CR1, were broadly expressed, thereby implying their potentially central role in cell trafficking (8). Furthermore, Zhaocheng Dong and his colleagues investigated the differences in molecular mechanisms and key biomarkers between membranous nephropathy and LN. They screened out six hub genes (IFI6, MX1, XAF1, HERC6, IFI44L, and IFI44) between the biopsy samples of these two nephritises (9). Meanwhile, Andrea Fava et al. analyzed the patterns of 1000 urine protein biomarkers in 30 patients with active LN. They identified an interferon-γ response gradient in LN (10). Studies focusing on renal involvement in patients with SLE mainly used renal biopsy or urine. However, analysis concerning whole blood samples was limited. As we all know, blood sample is easy to obtain and the DEGs in blood from indicated groups could offer information concerning disease pathogenesis. Moreover, identified DEGs can stratify patients with different organ involvement. Therefore, biomarkers in blood are of great value in identifying high risk patients with renal involvement. Through the combination of microarray and bioinformatics analyses, exploring potential key genes and pathway networks that are closely related to renal involvement is possible.

The two datasets including in our study was GSE49454 and GSE65391. The previous studies concerning these two datasets mainly focusing on detecting possible pathogenesis of SLE. The original article about GSE49454 revealed that complex interferon (IFN) signatures in SLE, which are not restricted to the previous IFNα signature, but which also involve IFNβ and IFNγ (11). In addition, GSE65391 also discovered a prevalent IFN signature and identified a plasma blast signature as the most robust biomarker of disease activity (12). However, both studies did not analyze the key genes related to active renal involvement, which is the most often and most severe complication, in patients with SLE. In this study, we used bioinformatics approaches to screen for biomarkers for active renal involvement in patients with SLE. In addition, the transcriptional factors (TFs) were predicted by database search and a TF-message RNA network was constructed. These results may guide future experimental research and clinical transformation.

## Materials and methods

### Data collection

**FIGURE 1:**
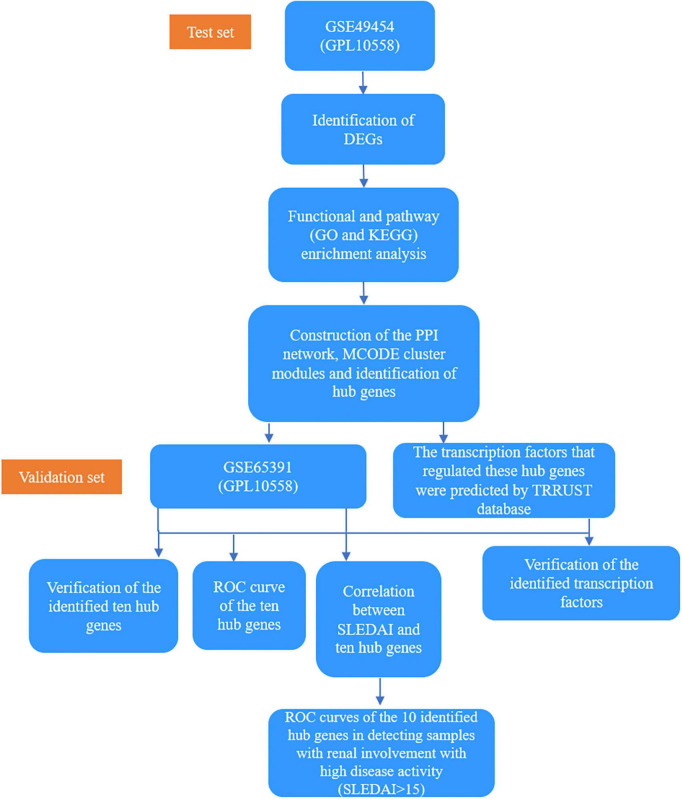
A flowchart of the overall study.

“Systemic lupus erythematosus” was used as the keyword to search for expression profiling of SLE in the Gene Expression Omnibus (GEO) database, which is a public repository database (13). Studies that met the following criteria were included, as follows: (1) whole genome expression data of SLE, (2) datasets containing more than five samples, and (3) datasets containing renal involvement information about the samples. Finally, one dataset GSE49454 (GPL10558), which included 64 active renal involvement samples and 93 without active renal involvement samples, was selected as the test set (11). One dataset GSE65391 (GPL10558), which included 69 active renal involvement samples and 68 without active renal involvement samples, was selected as the validation set (12). Active renal involvement was defined by the presence of at least one component of the renal SLEDAI, including urinary casts, hematuria, proteinuria, and pyuria. Samples with hematuria attributable to menstruation were excluded. In GSE49454 dataset, “renal: Y” was used to indicate active renal involvement. In GSE65391, “renal: 1” was used to indicate active renal involvement. Their basic details are listed in Table 1 and the basic information of our test set, GSE49454 is shown in Supplementary Table 1. A total of 86 patients in GSE65391 underwent renal biopsy. Meanwhile, 47 patients did not have renal biopsy at the time of the visit, which recorded as “no-LN.” The histopathological patterns, including membranous, proliferative, and non-proliferative, of 86 patients in GSE65391 were recorded. The histopathological patterns of four patients in GSE65391 were not available in the dataset. The detailed clinical information of GSE65391 is listed in Table 2 and Supplementary Figure 1. The overall flowchart of this study is shown in Figure 1.

**TABLE 1:** Information for selected microarray datasets.

GEO accession	Platform	Samples		Source tissue	SLE patients		Attribute	Diagnostic criteria
		SLE	HC		Active renal involvement	Without active renal involvement		
GSE49454	GPL10558	157	20	Whole blood	64	93	Test set	1997 ACR criteria for SLE (42)
GSE65391	GPL10558	137	53	Whole blood	69	68	Validation set	Not mentioned

Active renal involvement: defined by the presence of at least one component of the renal SLEDAI.

**TABLE 2:** Detailed clinical information of GSE65391.

	Non-LN	Membranous	Proliferative	Non-proliferative
Age (mean ± SD)	14.26 ± 2.67	15.14 ± 1.67	13.59 ± 2.86	13.19 ± 3.68
Sex (female/male)	42/5	7/2	54/10	12/1
Number of patients with active renal involvement	11	8	43	3
SLEDAI (mean ± SD)	5.77 ± 4.21	7.44 ± 1.51	12.14 ± 8.51	7.54 ± 8.41
Number of patients been biopsied at first visit	0	4	39	7
Days since kidney biopsy (mean ± SD)	-	450 ± 710.47	528.54 ± 704.71	1054.86 ± 1462.02

LN: lupus nephritis; Active renal involvement: defined by the presence of at least one component of the renal SLEDAI.

### Identification of differentially expressed genes

The raw expression data of GSE49454 were analyzed. The DEGs between patients with active renal involvement and those without active renal involvement were obtained through the online web-based tool GEO2R. An adjusted *P* value < 0.05 was considered statistically significant. The graphs of heatmap, Uniform Manifold Approximation, and Projection (UMAP) and Principal Component Analysis (PCA) were analyzed and visualized by RStudio^1^. The package used for UMAP was Umap (version 0.2.7.0), and the package used for PCA was Stats (version 3.6.0).

### Functional enrichment analysis

Gene Ontology (GO) and Kyoto Encyclopedia of Genes and Genomes (KEGG)^2^ enrichment analyses for the identified DEGs were performed by R packages (clusterProfile, ggplot2, and GOplot) (14). The ClusterProfile package was used to analyze the DEGs. The Ggplot2 and GOplot packages were used to visualize the results.

### Construction of protein–protein interaction network and identification of hub genes

The DEGs were analyzed by using the online tool STRING^3^ to construct the PPI network. The cut-off standard was set as a combined score >0.4 (15). Then, the results were visualized by CytoScape software. Molecular Complex Detection (MCODE) V1.5.1, which is a plug-in of CytoScape, was used to identify significant modules (MCODE score ≥4) (16). GO and KEGG analyses were also used for the identified modules. Moreover, the hub genes were selected using CytoHubba, which is another plug-in of Cytoscape, according to the number of associations with other genes in the PPI network (17). Seven common algorithms [Maximum Neighborhood Component (MNC), Density of Maximum Neighborhood Component (DMNC), Maximal Clique Centrality (MCC), Degree, Closeness, Radiality, and Stress] were used in evaluating and selecting hub genes.

### Prediction of transcription factors

Transcriptional Regulatory Relationships Unraveled by Sentence Based Text Mining (TRRUST), a database for the prediction of transcriptional regulatory networks, was used in predicting TFs that regulate hub genes, and an adjusted *P* value of <0.05 was considered significant (18).

### Statistical analysis

Statistical analysis was performed with Rstudio software and IBM SPSS Statistics 22 (SPSS, Inc., Chicago, IL, USA). Continuous variables were presented as the mean ± standard deviation (SD). The expression levels of the identified hub genes were validated by GSE65391 using Mann–Whitney *U* test, as the samples do not satisfy the normality test. The area under the curve of the receiver operating characteristic curve (ROC-AUC) was used to compare the diagnostic performance of different hub genes. Linear correlation analysis was performed by the software GraphPad Prism 7 to determine the relationship between SLE disease activity index (SLEDAI) and the expression levels of the identified hub genes. Pearson correlation coefficient was used to calculate the correlation coefficients.

## Results

### Identification of common differentially expressed genes

**FIGURE 2:**
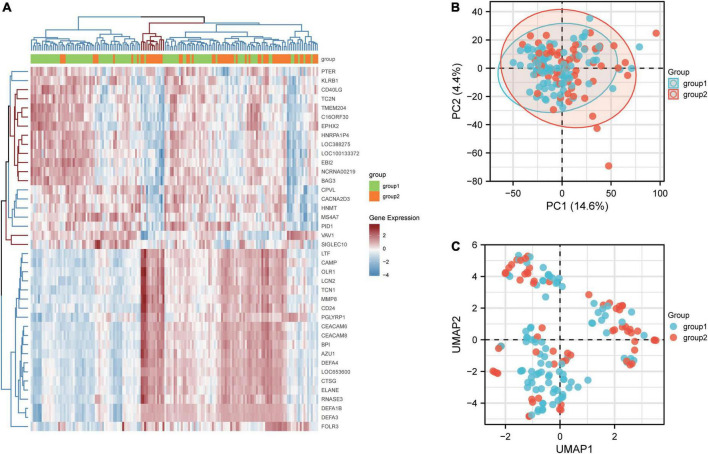
Identification of DEGs between patients with and without renal involvement. **(A)** Heatmap of GSE65391, **(B)** principal component analysis (PCA) plot generated from DEGs in GSE49454, and **(C)** uniform Manifold Approximation and Projection (UMAP) showing distinct clusters of DEGs in GSE49454. Group1 stands for the patients without active renal involvement and group2 stands for patients with active renal involvement. Data points in red represent upregulated genes, and those in blue represent downregulated genes.

By analyzing the differences between patients with active renal involvement and those without active renal involvement with two-group comparison, 182 DEGs from GSE49454 were identified. DEGs with adj. *P* value <0.05 were first screened out and the expression of top20 genes with highest and lowest expression was visualized in heatmap, which is shown in Figure 2A. The top 20 genes with highest and lowest expression in patients with renal involvement and without renal involvement were clustered on the heat map respectively. The logFC value and adjusted P value of the identified182 DEGs in GSE65391 were listed in Supplementary Table 2. The PCA and UMAP are shown in Figures 2B,C. Group1 stands for the patients without active renal involvement and group2 stands for patients with active renal involvement. PCA demonstrated that variations were represented by active renal involvement and without active renal involvement in GSE49454 for 4.4% and 14.6% respectively. In addition, Figure 2C presents the UMAP of GSE49454. However, there is not good discrimination in either the PCA or UMAP analysis, indicating that the difference between samples can be explained by PCA map and UMAP is limited.

### Biological functions analyses, protein–protein interaction network construction, and molecular complex detection cluster module identification

**FIGURE 3:**
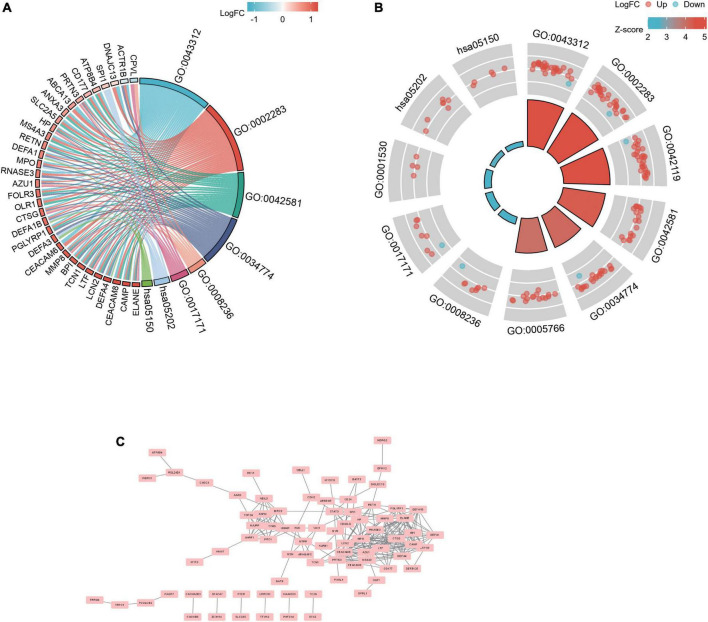
PPI network and functional enrichment of DEGs. The enrichment analysis results of GO and KEGG pathway [**(A)**: chordal graph; and **(B)**: loop graph]. Adjusted *P* value < 0.05 was considered significant. **(C)** The interaction network between proteins coded by DEGs.

Gene ontology and KEGG analyses were used for analyzing the 182 common DEGs (Figures 3A,B) (19–21). Based on GO enrichment, the biological process acted primarily on neutrophil degranulation, neutrophil activation involved in immune response, and neutrophil activation. These proteins were primarily located in specific granule, secretory granule lumen, and primary lysosome. For molecular functions, the proteins played roles in serine-type peptidase activity, serine hydrolase activity, and lipopolysaccharide binding. According to KEGG pathway analysis, these proteins were primarily involved in transcriptional misregulation in cancer and *Staphylococcus aureus* infection (Table 3). The PPI network for the 182 DEGs was constructed after the common DEGs were imported to STRING (Figure 3C).

**TABLE 3:** GO and KEGG analysis of DEGs.

Ontology	ID	Description	GeneRatio	BgRatio	*p*.adjust
BP	GO:0043312	Neutrophil degranulation	30/123	485/18670	6.45e-18
BP	GO:0002283	Neutrophil activation involved in immune response	30/123	488/18670	6.45e-18
BP	GO:0042119	Neutrophil activation	30/123	498/18670	6.45e-18
CC	GO:0042581	Specific granule	19/129	160/19717	1.95e-16
CC	GO:0034774	Secretory granule lumen	21/129	321/19717	2.37e-13
CC	GO:0005766	Primary lysosome	16/129	155/19717	2.37e-13
MF	GO:0008236	Serine-type peptidase activity	8/122	182/17697	0.007
MF	GO:0017171	Serine hydrolase activity	8/122	186/17697	0.007
MF	GO:0001530	Lipopolysaccharide binding	4/122	35/17697	0.010
KEGG	hsa05202	Transcriptional misregulation in cancer	7/60	192/8076	0.052
KEGG	hsa05150	*Staphylococcus aureus* infection	5/60	96/8076	0.052

BP, biological process; CC, cellular component; MF, molecular function; KEGG, kyoto encyclopedia of genes and genomes.

**FIGURE 4:**
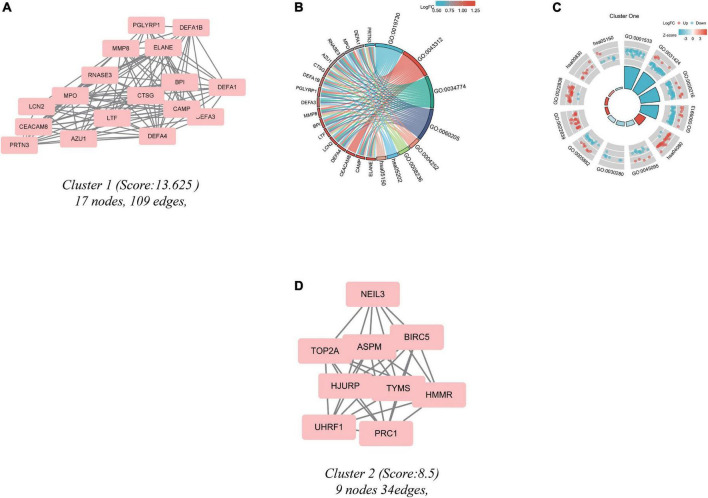
Cluster modules extracted by MCODE and enrichment analysis of the modular genes. **(A,D)** Significant gene clustering modules. **(B,C)** GO and KEGG enrichment analysis of the first modular genes. Two cluster modules extracted by MCODE. Cluster 1 **(A)** had the higher cluster score (MCODE score = 13.625), followed by cluster 2 **(D)** (MCODE score = 8.5). Adjusted *P* value < 0.05 was considered significant.

Significant modules of the PPI network were identified by MCODE. An MCODE score of 4 was set as a threshold. Two modules with MCODE scores of ≥4 are illustrated in Figure 4. One cluster (MCODE score = 13.625) had 17 nodes and 109 edges (Figure 4A). GO analysis showed that the proteins in the cluster were related to keratinization, keratinocyte differentiation, and epidermal cell differentiation (Figures 4B,C). KEGG pathway analysis showed that these proteins were primarily involved in neuroactive ligand-receptor interaction, retinol metabolism, and *S. aureus* infection (Figures 4B,C). The other module (MCODE score = 8.5) had 9 nodes and 34 edges (Figure 4D). Since the logFC of the DEGs in cluster 2 were not substantial, the enrichment result may have bias.

### Selection and analysis of hub genes

**FIGURE 5:**
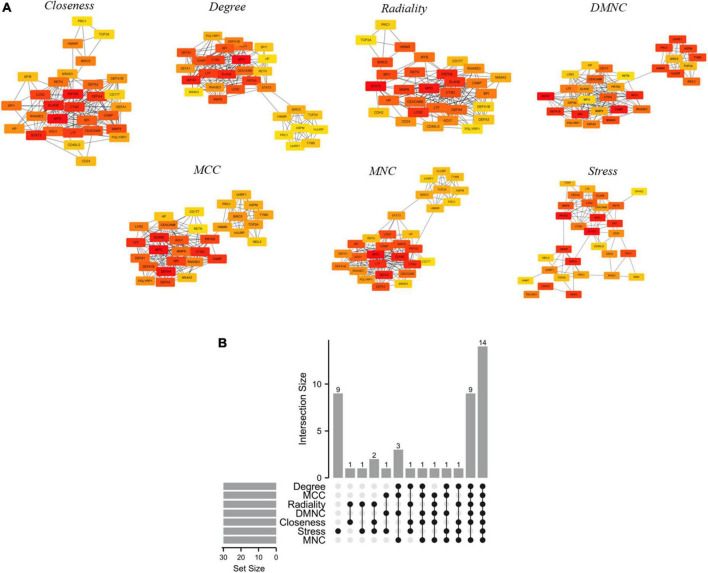
Hub genes identified by different algorithms and UpSet diagram. **(A)** Hub gene identified by seven different algorithms (Degree, MNC, DMNC, MCC, Closeness, Radiality, and Stress) using Cytohubba. Node color reflects the degree of connectivity. Red represents a higher degree, and yellow represents a lower degree. **(B)** The UpSet diagram showed that the seven algorithms screened 14 overlapping hub genes.

PPI is a useful way for presenting many types of biological data. We can measure nodes by their network features to infer their importance in the network, and it can help us identify central elements of biological networks. CytoHubba provides different topological analysis methods including Degree, MNC, DMNC, MCC, Closeness, Radiality, and Stress based on shortest paths (17). A hub gene is defined as a gene that plays a critical role in biological processes and is often influenced by the regulation of other genes in related pathways. Therefore, hub genes are often an important action target and a hot area of research. The top 30 hub genes were calculated using the abovementioned seven algorithms of the plug-in CytoHubba (Figure 5A). The red ones represented high scores and yellow ones represented low scores. After the determination of the intersection of the UpSet diagram, 14 common hub genes were discovered, namely, defensin alpha 4 (DEFA4), cathepsin G (CTSG), resistin (RETN), CEA cell adhesion molecule 8 (CEACAM8), proteinase 3 (PRTN3), DNA topoisomerase II alpha (TOP2A), lactotransferrin (LTF), protein regulator of cytokinesis 1 (PRC1), myeloperoxidase (MPO), elastase, neutrophil expressed (ELANE), matrix metallopeptidase 8 (MMP8), baculoviral IAP repeat containing 5 (BIRC5), hyaluronan mediated motility receptor (HMMR), and lipocalin 2 (LCN2,also known as NGAL; Figure 5B). Table 4 shows the GO and KEGG analysis of the 14 common hub genes. According to GO enrichment, the biological process acted mainly on neutrophil degranulation, neutrophil activation involved in immune response, and neutrophil activation, and these proteins were mainly located in secretory granule lumen, cytoplasmic vesicle lumen, and vesicle lumen. As to molecular functions, these proteins mainly took part in serine-type endopeptidase activity, serine-type peptidase activity, and serine hydrolase activity. Meanwhile, KEGG pathway analysis presented that these proteins were mainly involved in transcriptional misregulation in cancer, platinum drug resistance, and SLE.

**TABLE 4:** GO and KEGG analysis of 14 common hub genes.

Ontology	ID	Description	*p*.adjust	Gene ID
BP	GO:0043312	Neutrophil degranulation	1.96e-11	CEACAM8/CTSG/DEFA4/ELANE/LCN2/LTF/MMP8/MPO/PRTN3/RETN
BP	GO:0002283	Neutrophil activation involved in immune response	1.96e-11	CEACAM8/CTSG/DEFA4/ELANE/LCN2/LTF/MMP8/MPO/PRTN3/RETN
BP	GO:0042119	Neutrophil activation	1.96e-11	CEACAM8/CTSG/DEFA4/ELANE/LCN2/LTF/MMP8/MPO/PRTN3/RETN
CC	GO:0034774	Secretory granule lumen	3.80e-12	CTSG/DEFA4/ELANE/LCN2/LTF/MMP8/MPO/PRTN3/RETN
CC	GO:0060205	Cytoplasmic vesicle lumen	3.80e-12	CTSG/DEFA4/ELANE/LCN2/LTF/MMP8/MPO/PRTN3/RETN
CC	GO:0031983	Vesicle lumen	3.80e-12	CTSG/DEFA4/ELANE/LCN2/LTF/MMP8/MPO/PRTN3/RETN
MF	GO:0004252	Serine-type endopeptidase activity	4.21e-06	CTSG/ELANE/LTF/MMP8/PRTN3
MF	GO:0008236	Serine-type peptidase activity	4.21e-06	CTSG/ELANE/LTF/MMP8/PRTN3
MF	GO:0017171	Serine hydrolase activity	4.21e-06	CTSG/ELANE/LTF/MMP8/PRTN3
KEGG	hsa05202	Transcriptional misregulation in cancer	0.013	DEFA4/ELANE/MPO
KEGG	hsa01524	Platinum drug resistance	0.021	BIRC5/TOP2A
KEGG	hsa05322	Systemic lupus erythematosus	0.047	CTSG/ELANE

BP, biological process; CC, cellular component; MF, molecular function; KEGG, kyoto encyclopedia of genes and genomes.

### Validation of hub genes expression in GSE65391

**FIGURE 6:**
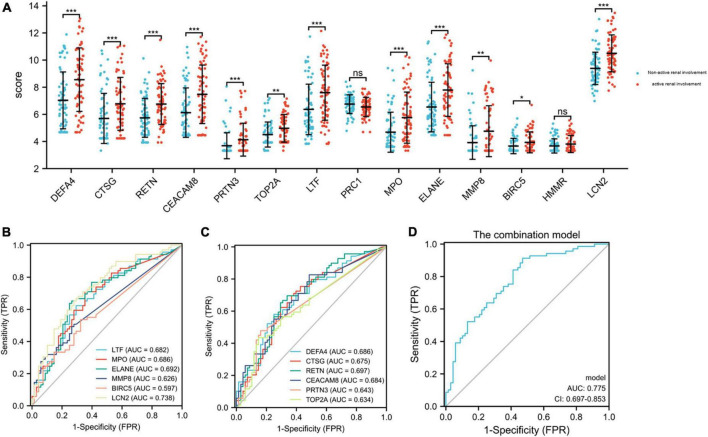
Expression level of hub genes in GSE65391 and ROC curves of the 12 identified and verified hub genes. **(A)** The verification of hub genes in GSE65391. The comparison between the two sets of data with the mean T test. Except PRC1 and HMMR, the expression levels of other 12 hub genes were verified in GSE65391. **(B,C)** The ROC curves of the 12 hub genes. **(D)** The ROC curve of the combination model of the 12 hub genes. RStudio (https://www.R-project.org) was used in statistical analysis. *P* value < 0.05 was considered statistically significant. *P < 0.05; **P < 0.01; ***P < 0.001.

The GSE65391 dataset was used to verify the expression of the identified hub genes. The expression levels of DEFA4, CTSG, RETN, CEACAM8, PRTN3, TOP2A, LTF, MPO, ELANE, MMP8, BIRC5, and LCN2 (also known as NGAL) were significantly increased in the active renal involvement samples compared with those without active renal involvement samples (*P* < 0.05, Figure 6A).

### Receiver operating characteristic curves of 12 verified hub genes in renal involvement samples

The series matrix file of GSE65391 that offers the different expression levels of the identified hub genes was imported into the RStudio. The software calculated the sensitivity, specificity, cut-off value, and AUC of the 12 verified hub genes (Table 5). LCN2 (also known as NGAL) has a certain diagnostic accuracy with the AUC over 0.7 (Figures 6B,C). The combination model of the 12 hub genes has a certain diagnostic accuracy in detecting active renal involvement patients among SLE patients (Figure 6D).

**TABLE 5:** The sensitivity, specificity, and AUC of the 12 verified hub genes in detecting renal involvement in SLE.

Rank	Gene symbol	Sensitivity (%)	Specificity (%)	AUC (95% CI)	Cut-off value
1	DEFA4	58	75	0.686 (0.598-0.775)	8.063
2	CTSG	62.3	70.6	0.675 (0.585-0.766)	6.103
3	RETN	65.2	70.6	0.697 (0.609-0.786)	6.219
4	CEACAM8	82.7	51.5	0.684 (0.595-0.773)	5.418
5	PRTN3	47.8	83.8	0.643 (0.563-0.722)	3.623
6	TOP2A	43.5	85.3	0.634 (0.543-0.726)	5.263
7	LTF	62.3	70.6	0.682 (0.592-0.772)	6.846
8	MPO	71	64.7	0.686 (0.597-0.775)	4.547
9	ELANE	63.8	75	0.692 (0.602-0.782)	7.053
10	MMP8	49.3	73.5	0.626 (0.544-0.709)	3.666
11	BIRC5	53.6	67.6	0.597 (0.508-0.685)	3.476
12	LCN2	65.2	72.1	0.738 (0.654-0.821)	9.888
	Model	91.3	52.9	0.775 (0.697-0.853)	−0.65

AUC, area under the curve; CI, confidence interval. Combination model: −10.9192 + 0.2482 * DEFA4 + −0.159 * CTSG + 0.2673 * RETN + −0.2245 * CEACAM8 + −0.5405 * PRTN3 + −0.3169 * LTF + 0.4004 * MPO + −0.1196 * ELANE + 1.1 * LCN2 + −0.1195 * MMP8 + 0.3778 * BIRC5 + 0.2318 * TOP2A.

### Correlation between SLE disease activity index and different hub genes in GSE65391

**FIGURE 7:**
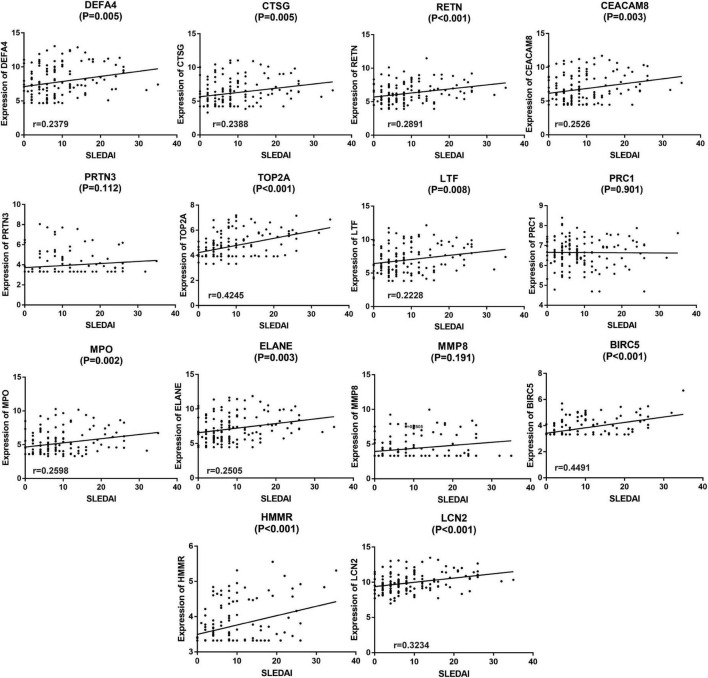
Linear correlation analysis of the 14 hub genes and SLEDAI. The expression of 10 hub genes (except PRTN3, PRC1, MMP8, and HMMR) were discovered to be linear related to SLEDAI and validated by GSE65391. *P* < 0.05 was considered statistically significant.

Since the active renal involvement was defined by the presence of at least one component of the renal SLEDAI, linear correlation analysis was performed to clarify the relationship between SLEDAI and the expression of different hub genes. The results are shown in Figure 7. In the analysis process, 11 of the 14 hub genes, namely, DEFA4, CTSG, RETN, CEACAM8, TOP2A, LTF, MPO, ELANE, BIRC5, HMMR, and LCN2 (also known as NGAL), were statistically positively associated with SLEDAI (*P* < 0.05, Figure 7). Since the expression of HMMR was not validated by GSE65391, 10 genes which were validated and positively related with SLEDAI were included in the following analyses.

### Receiver operating characteristic curves of the 10 identified hub genes in detecting samples with active renal involvement and high disease activity (SLEDAI > 15)

**FIGURE 8:**
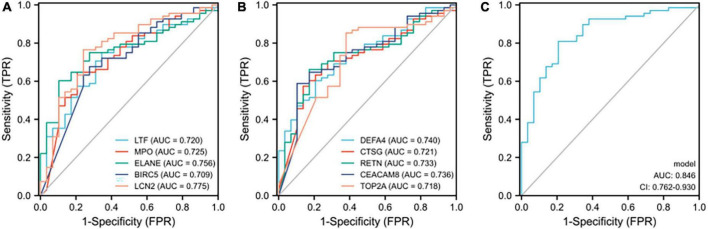
ROC curves of the 10 hub genes in identifying renal involvement patients with high disease activity (SLEDAI > 15). **(A,B)** ROC curves of the 10 hub genes. **(C)** ROC curve of the combination model of the 10 hub genes. The combination model was 14.6627 + –0.3795 * DEFA4 + 0.2401 * CTSG + –0.0942 * RETN + –0.0114 * CEACAM8 + –0.2822 * TOP2A + 0.5422 * LTF + 0.1112 * MPO + –0.1143 * ELANE + –0.8064 * BIRC5 + –1.0725 * LCN2. The AUC of the combination model was 0.846 and the 95% confidence interval (CI) was 0.762-0.930.

As 10 of the 14 hub genes were statistically positively associated with SLEDAI and active renal involvement stands for the presence of at least one component of the renal SLEDAI, we further examined the diagnostic ability in identifying samples with active renal involvement and high disease activity (SLEDAI > 15). All 10 hub genes had a certain diagnostic accuracy with AUC values of over 0.7 (Figures 8A,B). The combination model of the 10 hub genes had a certain diagnostic accuracy (AUC = 0.846) in detecting patients with renal involvement and with high disease activity (SLEDAI > 15, Figure 8C). The sensitivity, specificity, cut-off value, and AUC of the 10 hub genes are listed in Table 6. The combination model was 14.6627 + −0.3795 * DEFA4 + 0.2401 * CTSG + −0.0942 * RETN + −0.0114 * CEACAM8 + −0.2822 * TOP2A + 0.5422 * LTF + 0.1112 * MPO + −0.1143 * ELANE + −0.8064 * BIRC5 + −1.0725 * LCN2.

**TABLE 6:** The sensitivity, specificity, and AUC of the identified hub genes in detecting renal involvement patients with SLEDAI > 15.

Rank	Gene symbol	Sensitivity (%)	Specificity (%)	AUC (95% CI)	Cut-off value
1	DEFA4	60.3	79.3	0.740 (0.638-0.841)	7.17
2	CTSG	57.4	86.2	0.721 (0.614-0.828)	5.348
3	RETN	66.2	82.8	0.733 (0.627-0.839)	5.997
4	CEACAM8	58.8	89.7	0.736 (0.627-0.844)	6.07
5	TOP2A	85.3	62.1	0.718 (0.605-0.830)	5.286
6	LTF	75	65.5	0.720 (0.611-0.828)	7.244
7	MPO	64.7	79.3	0.725 (0.613-0.836)	4.547
8	ELANE	60.3	89.7	0.756 (0.658-0.854)	6.378
9	BIRC5	67.6	72.4	0.709 (0.597-0.821)	3.49
10	LCN2	76.5	75.9	0.775 (0.669-0.881)	10.074
	Model	80.9	79.3	0.846 (0.762-0.930)	0.699

SLEDAI, systemic lupus erythematosus disease activity index; AUC, area under the curve; CI, confidence interval. Combination model: 14.6627 + −0.3795 * DEFA4 + 0.2401 * CTSG + −0.0942 * RETN + −0.0114 * CEACAM8 + −0.2822 * TOP2A + 0.5422 * LTF + 0.1112 * MPO + −0.1143 * ELANE + −0.8064 * BIRC5 + −1.0725 * LCN2.

### Prediction and verification of transcriptional factors

**FIGURE 9:**
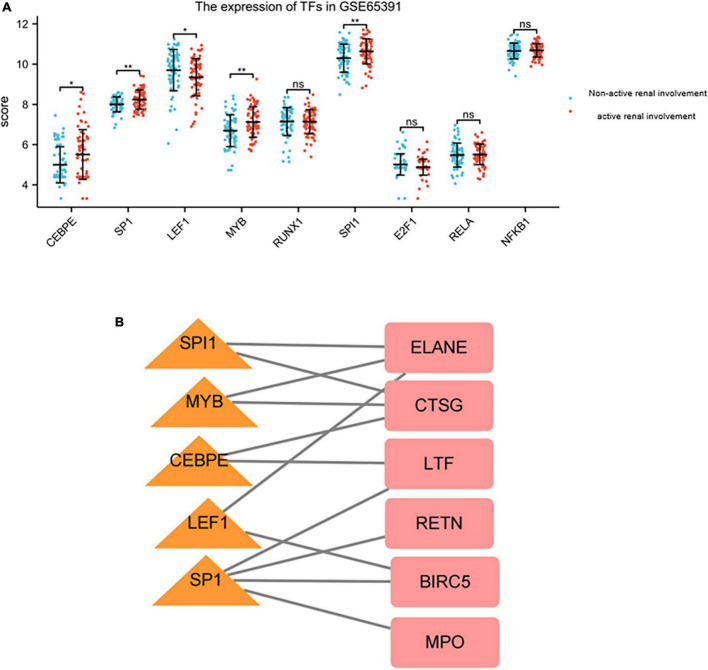
The expression of TFs in GSE65391 and TFs regulatory network. **(A)** The expression level of TFs in GSE65391. The expression of five TFs, namely SPI1, MYB, CEBPE, LEF1, and SP1 were validated by GSE65391. *P < 0.05, **P < 0.01. **(B)** The verified TF regulatory network. TFs were marked in yellow triangle, and the hub genes were marked in red rectangle.

Nine TFs that may regulate the expression of the hub genes were identified on the basis of the TRRUST database (Table 7). CCAAT/enhancer binding protein (C/EBP), epsilon (CEBPE), Sp1 transcription factor (SP1), lymphoid enhancer-binding factor 1 (LEF1), v-myb myeloblastosis viral oncogene homolog (avian) (MYB), runt-related transcription factor 1 (RUNX1), spleen focus forming virus (SFFV) proviral integration oncogene spi1 (SPI1), E2F transcription factor 1 (E2F1), v-rel reticuloendotheliosis viral oncogene homolog A (avian) (RELA), and nuclear factor of kappa light polypeptide gene enhancer in B-cells 1 (NFKB1) were predicted to have the capability to regulate six hub genes (LTF, CTSG, MPO, BIRC5, RETN, and ELANE) by acting as TFs. During further verification, the expression levels of five TFs, including CEBPE, SP1, LEF1, MYB, and SPI1, significantly changed between patients with renal involvement and those without renal involvement (*P* < 0.05, Figure 9A). The constructed network of TFs regulating message RNA is shown in Figure 9B.

**TABLE 7:** Key transcriptional factors (TFs) of hub genes.

Key TF	Description	*P*-value	List of overlapped genes
CEBPE	CCAAT/Enhancer binding protein (C/EBP), epsilon	5.30E-06	LTF, CTSG
SP1	Sp1 transcription factor	7.20E-05	MPO, BIRC5, RETN, LTF
LEF1	Lymphoid enhancer-binding factor 1	0.000102	BIRC5, ELANE
MYB	V-myb myeloblastosis viral oncogene homolog (avian)	0.000167	ELANE, CTSG
RUNX1	Runt-related transcription factor 1	0.000195	MPO, ELANE
SPI1	Spleen focus forming virus (SFFV) proviral integration oncogene spi1	0.00047	ELANE, CTSG
E2F1	E2F transcription factor 1	0.00217	BIRC5, TOP2A
RELA	V-rel reticuloendotheliosis viral oncogene homolog A (avian)	0.0105	BIRC5, LCN2
NFKB1	Nuclear factor of kappa light polypeptide gene enhancer in B-cells 1	0.0106	LCN2, BIRC5

## Discussion

The main purpose of our study is to identify the key genes related to active renal involvement in patients with SLE. A total of 182 DEGs were detected between patients with active renal involvement and those without active renal involvement. This study is a re-analysis of previous existed GEO datasets. The previous two study mainly focused on detecting possible pathogenesis of SLE (11, 12). However, both studies did not analyze the key genes related to active renal involvement, which is the most often and most severe complication, in patients with SLE. Therefore, we performed this study on the base of the two datasets. Of the DEGs detected, 14 were hub genes and 12 were verified by using the GSE65391 dataset. GO enrichment analysis revealed that the DEGs were significantly enriched in neutrophil degranulation, neutrophil activation that is involved in immune response, and neutrophil activation. Moreover, 10 hub genes, namely, DEFA4, CTSG, RETN, CEACAM8, TOP2A, LTF, MPO, ELANE, BIRC5, and LCN2 (also known as NGAL), were statistically positive related to SLEDAI and were able to detect patients with active renal involvement who had high disease activity (SLEDAI > 15). Moreover, a TF-message RNA network was constructed on the basis of database searching and verification by another dataset.

Neutrophils are key effector cells of innate immunity that are rapidly recruited to defend the host against invading pathogens. Neutrophils may kill pathogens by degranulation and through the release of neutrophil extracellular traps. After cell activation by different stimuli, granule contents are released into the phagosome or in the extracellular space through degranulation (22). Neutrophil-derived reactive oxygen species and granule proteases are implicated in the damage to and destruction of host tissues in the vascular tissue of SLE patients (23). In addition, accumulating evidence showed that dysregulated neutrophil activation contributes to SLE pathogenesis. According to our results, neutrophil degranulation and activation were upregulated in active renal involvement patients with SLE. Therefore, stabilizing the function of neutrophil may be a novel therapeutic strategy.

Furthermore, eight hub genes that may play roles in neutrophil degranulation and activation were detected, namely, CEACAM8, CTSG, DEFA4, ELANE, LCN2 (also known as NGAL), LTF, MPO, and RETN. The expressions of these eight hub genes increased in patients with active renal involvement; thus, the inhibition of these genes is a potential treatment option. CEACAM8, one of the cell adhesion molecules, is stored in specific neutrophils granules and is an activation marker of rapid neutrophils degranulation because of its increased expression in stimulated neutrophils (24). A previous study described a novel mechanism by which a natural danger-associated molecular pattern, with inflammatory properties in SLE, induces soluble CEACAM8 secretion (25). Defensins are a family of antimicrobial peptides of innate immunity with immunomodulatory properties. DEFA4, one of the members of defensins, is found in the granules of neutrophils and exhibits neutrophil α-defensin function (26). LTF, found in the secondary granules of neutrophils, is an important component of the non-specific immune system (27). The elevation of LTF in patients with renal involvement may result from the abnormal function of neutrophil degranulation and activation. LCN2 (also known as NGAL), a member of the lipocalin family, has a hydrophobic pocket that binds lipophilic molecules and is stored in human neutrophil granules. The upregulation of LCN2 was recently reported to correlate with proteinuria and renal flares in patients with SLE (28). Moreover, Weiwei Chen et al. proved that LCN2 is involved in LN development and acts as a driver of extraordinary expansion of Th1 cells (29). Therefore, targeting these four hub genes may have great potential in controlling active renal involvement in patients with SLE. ELANE and CTGS function as proteases during neutrophil degranulation and activation. When ELANE is activated, this protease hydrolyzes proteins within specialized neutrophil lysosomes called azurophil granules, as well as proteins of the extracellular matrix (30). CTGS may participate in the killing and digestion of engulfed pathogens and in connective tissue remodeling at inflammation sites (31). These two hub genes both play essential roles in neutrophil degranulation and activation and would be promising treatment targets. In addition, our study identified two hub genes which work as autoantigens in anti-neutrophil cytoplasmic antibody (ANCA)-associated vasculitis abnormally elevated in active renal involvement patients, including MPO and PRTN3. MPO stimulation of NETosis, a program for formation of neutrophil extracellular traps (NETs), which consist of modified chromatin decorated with bactericidal proteins from granules and cytoplasm, is one intriguing hypothesis for MPO directed pathogenicity (32, 33). Persistence of NET burden is associated with LN as well as elevated dsDNA antibodies and antiNET antibodies (34). PRTN3 encodes proteinase-3, which is another important autoantigens in ANCA-associated vasculitis. It enables to enzyme binding activity and involved it neutrophil extravasation process (35). Recently, a complement regulator C4BP was proved to limit the development of LN via inhibition of PRTN3 to significant downregulate neutrophils activity, indicating the possible link between ANCA-associated vasculitis and LN (36).

Our study also predicted the TFs of identified hub genes. Nine TFs were predicted to regulate eight hub genes. The expression of five TFs were validated by GSE65391. SPI1 is an Ets family transcription factor that is essential for lymphoid and myeloid development. A previous study demonstrated that the SNP in the 3-UTR of SPI1 is associated with elevated SPI1 mRNA level and with susceptibility to SLE (37). Meanwhile, SPI1 may participate in the pathogenesis of SLE (38). Our study detected that SPI1 was significantly upregulated in patients with renal involvement and SLE, thereby indicating its role in LN pathogenesis. CEBPE is essential for terminal differentiation and functional maturation of committed granulocyte progenitor cells. Aberrancies of immune cells in SLE can be traced back to the hematopoietic stem and progenitor cells associated with the abnormal function of CEBPE (39). SP1 is involved in many cellular processes and post-translational modifications as an activator or a repressor. An increasing amount of evidence demonstrates that SP1 plays an important regulatory role in the expression of several genes relevant to fibrosis (40). SP1 overexpression in the glomeruli of proliferative nephritis may be a result of the inflammatory process (41). SP1 was shown to be substantially elevated in patients with renal involvement. Current treatments are effective only in 30% of LN patients, thereby emphasizing the need for novel therapeutic strategies. Targeting these TFs to regulate the hub genes is promising in the future.

In conclusion, our study aimed to identify and verify hub genes and TFs that may serve as promising treatment targets for patients with active renal involvement in SLE. Ten genes were identified and verified as hub genes. The hub genes had a certain diagnostic accuracy in detecting patients with active renal involvement and high disease activity. GO and KEGG pathway enrichment analyses revealed that these genes were significantly enriched in neutrophil degranulation, neutrophil activation involved in immune response, and neutrophil activation. Moreover, five TFs were predicted to participate in the regulation of hub genes. The expressions of the five TFs were verified by another dataset. This study may guide future experimental research and clinical transformation.

## Data availability statement

The datasets presented in this study can be found in online repositories. The names of the repository/repositories and accession number(s) can be found in the article/Supplementary material.

## Author contributions

LX designed the study. LX and WX did data collection and wrote the manuscript. SL revised the manuscript. All authors read and approved the final manuscript.

## Footnotes

1
https://www.R-project.org

2
www.kegg.jp/kegg/kegg1.html

3
https://string-db.org
