# Supplementary material for: Potential biomarkers for active renal involvement in systemic lupus erythematosus patients
Source: Front Med (Lausanne). 2022 Dec 1;9:995103. doi: 10.3389/fmed.2022.995103 (PMC9754094; doi:10.3389/fmed.2022.995103)
Supplement: Supplementary file 1 [file Data_Sheet_1.docx]

| Supplementary table 1 the basic information of the test set (GSE49454) | | |
| --- | --- | --- |
|  | active renal involvement | without active renal involvement |
| age(mean±SD) | 40.09±9.69 | 41.24±12.91 |
| gender(female/male) | 49/15 | 82/11 |
| SLEDAI(mean±SD) | 12.34±5.75 | 3.62±3.06 |
| cyclophosphamide[n(%)] | 11(17.18%) | 1(1.07) |
| azathioprine[n(%)] | 11(17.18%) | 37(39.78%) |
| Mycophenolate mofetil [n(%)] | 11(17.18) | 13(13.98%) |
| cortisone[n(%)] | 64(100%) | 83(89.25%) |
| The dosage of cortisone(mean±SD,mg/d) | 25.46±18.49 | 8.51±5.15 |
| hydroxychloroquine[n(%)] | 37(57.81) | 53(56.99%) |
| The dosage of hydroxychloroquine(mean±SD,mg/d) | 216.13±192.64 | 193.55±182.86 |
| SD, standard deviation; SLEDAI, systemic lupus erythematosus disease activity index | | |

| Supplementary table 2 the log FC and adjusted P value of identified DEGs in GSE65391 | | | | | |
| --- | --- | --- | --- | --- | --- |
| Gene.Symbol | adj.P.Val | logFC | Gene.Symbol | adj.P.Val | logFC |
| LOC653600 | 0.016856 | 1.46971 | MTF1 | 0.012898 | 0.30346 |
| ELANE | 0.005073 | 1.250064 | VIPR1 | 0.012898 | -0.30326 |
| CAMP | 0.001887 | 1.240649 | CA5B | 0.020242 | -0.29982 |
| CEACAM8 | 0.007254 | 1.222089 | FTSJD1 | 0.015671 | -0.29929 |
| DEFA4 | 0.020242 | 1.194816 | DOCK9 | 0.034669 | -0.29921 |
| LCN2 | 0.001887 | 1.127679 | TRPM4 | 0.028074 | -0.2971 |
| LTF | 0.012898 | 1.066906 | LOC389816 | 0.015671 | -0.29613 |
| TCN1 | 0.001887 | 1.065125 | LEPROTL1 | 0.016293 | -0.29545 |
| BPI | 0.004579 | 1.063099 | ZNF702P | 0.019713 | -0.29408 |
| MMP8 | 0.003334 | 1.061734 | SH3BP5L | 0.036316 | 0.293183 |
| CEACAM6 | 0.017299 | 1.052376 | CACNB3 | 0.012441 | -0.29239 |
| DEFA3 | 0.033947 | 0.981891 | BIRC5 | 0.034315 | 0.288203 |
| PGLYRP1 | 0.00592 | 0.977978 | OPRL1 | 0.03461 | 0.286764 |
| DEFA1B | 0.034974 | 0.974536 | LOC283392 | 0.047467 | 0.286099 |
| CTSG | 0.028074 | 0.956641 | SPI1 | 0.015671 | 0.284817 |
| OLR1 | 0.002377 | 0.916337 | LOC729034 | 0.012067 | 0.284159 |
| FOLR3 | 0.005961 | 0.910172 | ASPM | 0.039315 | 0.283886 |
| CD24 | 0.001887 | 0.90813 | CASC3 | 0.037899 | 0.280711 |
| AZU1 | 0.008399 | 0.840187 | STAT3 | 0.018653 | 0.279089 |
| RNASE3 | 0.035683 | 0.819752 | ARHGAP5 | 0.046747 | 0.278493 |
| MPO | 0.016966 | 0.799596 | FADS1 | 0.02834 | -0.27836 |
| DEFA1 | 0.042109 | 0.781208 | PHF21A | 0.020688 | 0.277687 |
| RETN | 0.006595 | 0.747079 | LOC646836 | 0.015815 | -0.27419 |
| MS4A3 | 0.0238 | 0.74656 | NIF3L1 | 0.028537 | -0.27358 |
| COL17A1 | 0.005073 | 0.695661 | SLC25A43 | 0.041587 | -0.27303 |
| LOC554223 | 0.015671 | 0.688538 | TTYH2 | 0.046404 | -0.2727 |
| LOC100134379 | 0.017299 | 0.685822 | LOC400214 | 0.004579 | -0.27109 |
| HP | 0.025398 | 0.685814 | HJURP | 0.015671 | 0.269906 |
| TACSTD2 | 0.043075 | 0.682995 | NRD1 | 0.005073 | 0.266585 |
| LOC100008589 | 0.005073 | 0.662863 | APBB1IP | 0.012441 | 0.26457 |
| LOC100132394 | 0.004579 | 0.642089 | CDH2 | 0.04539 | -0.2645 |
| SLC2A5 | 0.003481 | 0.595571 | ZNF277 | 0.020242 | -0.26339 |
| ANXA3 | 0.034693 | 0.587714 | BATF3 | 0.034315 | -0.26243 |
| ABCA13 | 0.02167 | 0.577815 | TRPC1 | 0.03377 | -0.25587 |
| KLRB1 | 0.017299 | -0.52825 | KIAA0251 | 0.047175 | -0.25508 |
| CACNA2D3 | 0.004579 | -0.52713 | PAQR7 | 0.037899 | -0.25357 |
| LOC284648 | 0.020242 | 0.524759 | RHOC | 0.048994 | -0.2535 |
| PTPN20 | 0.005073 | 0.489005 | NME7 | 0.044538 | -0.24689 |
| C16ORF30 | 0.028537 | -0.48831 | SPATA7 | 0.015671 | -0.24644 |
| PRTN3 | 0.047467 | 0.479656 | HLA-F | 0.034974 | 0.246189 |
| FLJ41352 | 0.005961 | 0.469793 | RSL24D1 | 0.042029 | -0.24516 |
| HIST2H2AA3 | 0.010151 | 0.458426 | LDOC1 | 0.034315 | -0.24484 |
| TMEM204 | 0.017299 | -0.45691 | KIAA0513 | 0.033384 | 0.24035 |
| RRAGD | 0.005073 | 0.447208 | KCNT1 | 0.02834 | -0.23813 |
| EBI2 | 0.038001 | -0.44682 | KLF13 | 0.023377 | -0.23658 |
| LOC653061 | 0.02834 | 0.438605 | ZC3H14 | 0.017263 | -0.236 |
| HIST2H2AC | 0.015815 | 0.427919 | LOC728517 | 0.028537 | -0.22974 |
| HIST2H2BE | 0.023362 | 0.427881 | ZNF134 | 0.028537 | -0.22904 |
| TOP2A | 0.034315 | 0.42653 | AQP7P1 | 0.036705 | 0.227653 |
| MS4A7 | 0.042109 | -0.42278 | TMEM106C | 0.0238 | 0.226721 |
| KIAA0367 | 0.025398 | 0.42244 | STX2 | 0.049755 | -0.22663 |
| TYMS | 0.016293 | 0.420324 | RNF216 | 0.018403 | -0.22637 |
| LOC100133372 | 0.038001 | -0.41792 | PIWIL4 | 0.04539 | 0.224982 |
| LOC100134364 | 0.022133 | 0.415424 | MED12 | 0.012898 | 0.224549 |
| BAG3 | 0.015671 | -0.41447 | MGEA5 | 0.037899 | 0.223622 |
| HIST2H2AA4 | 0.020242 | 0.413576 | NDRG2 | 0.03377 | -0.22142 |
| CD177 | 0.028537 | 0.412829 | NLRP14 | 0.036316 | 0.219661 |
| PCOLCE2 | 0.041587 | 0.412662 | CBLL1 | 0.036705 | -0.21935 |
| LOC388275 | 0.048994 | -0.40534 | LOC729985 | 0.039315 | -0.21907 |
| SIGLEC10 | 0.0238 | -0.40457 | LOC401847 | 0.042109 | -0.21878 |
| CPVL | 0.038001 | -0.40137 | ANAPC7 | 0.049074 | -0.21852 |
| EPHX2 | 0.041868 | -0.39572 | NOL9 | 0.041868 | -0.21597 |
| PTER | 0.037899 | -0.3903 | LOC100128627 | 0.042952 | -0.21456 |
| HNRPA1P4 | 0.043676 | -0.38468 | HMMR | 0.047467 | 0.211913 |
| ATP8B4 | 0.015671 | 0.380716 | MYOCD | 0.023362 | 0.211304 |
| TC2N | 0.021601 | -0.3789 | PMPCB | 0.036316 | -0.21109 |
| HNMT | 0.024758 | -0.36917 | DEFB125 | 0.039349 | 0.210793 |
| MAP3K2 | 0.029408 | 0.36676 | TMEM42 | 0.03461 | -0.21022 |
| CD40LG | 0.042586 | -0.36347 | AAAS | 0.034315 | -0.20887 |
| TNCRNA | 0.0238 | 0.357241 | LOC646269 | 0.038197 | 0.204318 |
| LOC389827 | 0.03377 | 0.350815 | LCN1L1 | 0.046547 | -0.19676 |
| PLA2G4C | 0.04539 | 0.34601 | NAT9 | 0.043075 | -0.19291 |
| VAV1 | 0.023799 | -0.34299 | KIAA2013 | 0.037509 | 0.190481 |
| NCRNA00219 | 0.039348 | -0.34235 | NEIL3 | 0.036316 | 0.190416 |
| MYB | 0.024747 | 0.341548 | MAGMAS | 0.037899 | 0.188055 |
| PRC1 | 0.003755 | 0.338363 | FLJ20209 | 0.048994 | 0.187193 |
| UHRF1 | 0.005073 | 0.337785 | LEPREL1 | 0.046404 | -0.18686 |
| PID1 | 0.02834 | -0.33155 | LOC652685 | 0.038001 | -0.18573 |
| LOC651524 | 0.039348 | 0.330532 | LOC644898 | 0.017299 | -0.18539 |
| SPDYE1 | 0.022133 | 0.330312 | MYF5 | 0.028537 | 0.184996 |
| BEX4 | 0.047467 | -0.3293 | WDR31 | 0.039348 | 0.183626 |
| C11ORF46 | 0.019713 | -0.32585 | ACTR1B | 0.046747 | -0.18292 |
| TIGA1 | 0.046404 | -0.32132 | REV1 | 0.03377 | -0.18088 |
| ASAP1 | 0.012898 | 0.316289 | MIR488 | 0.039348 | -0.17867 |
| MTRR | 0.038197 | -0.31449 | ACO1 | 0.048994 | -0.17724 |
| LRRC8C | 0.042109 | -0.30745 | MMS19L | 0.037899 | -0.17677 |
| FAM134A | 0.033947 | 0.307398 | DNAJC13 | 0.048994 | 0.176168 |
| H2AFJ | 0.036316 | 0.30636 | MSN | 0.043075 | 0.167171 |
| OR4K15 | 0.038001 | 0.305613 | KIAA0922 | 0.022133 | 0.163409 |
| PLSCR3 | 0.043075 | -0.30505 | BRPF1 | 0.046404 | -0.16338 |
| LOC728787 | 0.041619 | 0.15675 | ATXN7L3 | 0.039693 | 0.160879 |
