# Supplementary figures and images for: Potential biomarkers for active renal involvement in systemic lupus erythematosus patients
Source: Front Med (Lausanne). 2022 Dec 1;9:995103. doi: 10.3389/fmed.2022.995103 (PMC9754094; doi:10.3389/fmed.2022.995103)

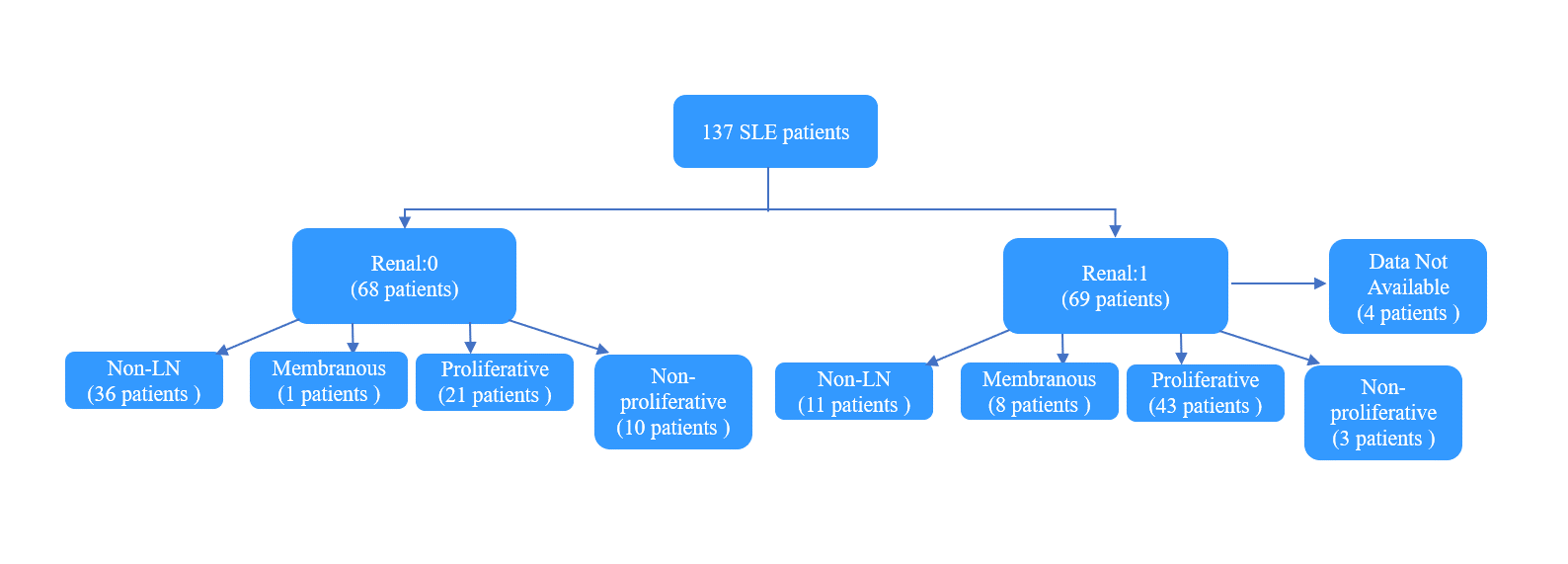

Supplement: Supplementary file 2 [file Image_1.png]
